# Spectral Signatures of Reorganised Brain Networks in Disorders of Consciousness

**DOI:** 10.1371/journal.pcbi.1003887

**Published:** 2014-10-16

**Authors:** Srivas Chennu, Paola Finoia, Evelyn Kamau, Judith Allanson, Guy B. Williams, Martin M. Monti, Valdas Noreika, Aurina Arnatkeviciute, Andrés Canales-Johnson, Francisco Olivares, Daniela Cabezas-Soto, David K. Menon, John D. Pickard, Adrian M. Owen, Tristan A. Bekinschtein

**Affiliations:** 1 Department of Clinical Neurosciences, University of Cambridge, Cambridge Biomedical Campus, Cambridge, United Kingdom; 2 Medical Research Council, Cognition and Brain Sciences Unit, Cambridge, United Kingdom; 3 Cambridge University Hospitals NHS Foundation Trust, Cambridge Biomedical Campus, Cambridge, United Kingdom; 4 Wolfson Brain Imaging Centre, University of Cambridge, Cambridge Biomedical Campus, Cambridge, United Kingdom; 5 Department of Psychology, University of California at Los Angeles, Los Angeles, California, United States of America; 6 Laboratory of Cognitive and Social Neuroscience, Universidad Diego Portales, Santiago, Chile; 7 Division of Anaesthesia, University of Cambridge, Cambridge Biomedical Campus, Cambridge, United Kingdom; 8 The Brain and Mind Institute, Natural Sciences Centre, The University of Western Ontario, London, Ontario, Canada; 9 Department of Psychology, University of Cambridge, Cambridge, United Kingdom; University of Pittsburgh, United States of America

## Abstract

Theoretical advances in the science of consciousness have proposed that it is concomitant with balanced cortical integration and differentiation, enabled by efficient networks of information transfer across multiple scales. Here, we apply graph theory to compare key signatures of such networks in high-density electroencephalographic data from 32 patients with chronic disorders of consciousness, against normative data from healthy controls. Based on connectivity within canonical frequency bands, we found that patient networks had reduced local and global efficiency, and fewer hubs in the alpha band. We devised a novel topographical metric, termed modular span, which showed that the alpha network modules in patients were also spatially circumscribed, lacking the structured long-distance interactions commonly observed in the healthy controls. Importantly however, these differences between graph-theoretic metrics were partially reversed in delta and theta band networks, which were also significantly more similar to each other in patients than controls. Going further, we found that metrics of alpha network efficiency also correlated with the degree of behavioural awareness. Intriguingly, some patients in behaviourally unresponsive vegetative states who demonstrated evidence of covert awareness with functional neuroimaging stood out from this trend: they had alpha networks that were remarkably well preserved and similar to those observed in the controls. Taken together, our findings inform current understanding of disorders of consciousness by highlighting the distinctive brain networks that characterise them. In the significant minority of vegetative patients who follow commands in neuroimaging tests, they point to putative network mechanisms that could support cognitive function and consciousness despite profound behavioural impairment.

## Author Summary

What are the neural signatures of consciousness? This is an elusive yet fascinating challenge to current cognitive neuroscience, but it takes on an immediate clinical and societal significance in patients diagnosed as vegetative and minimally conscious. In these patients, it leads us to ask whether we can test for the presence of these signatures in the absence of any external signs of awareness. Recent conceptual advances suggest that consciousness requires a dynamic balance between integrated and differentiated networks of information exchange between brain regions. Here we apply this insight to study such networks in patients and compare them to healthy adults. Using the science of graph theory, we show that the rich and diversely connected networks that support awareness are characteristically impaired in patients, lacking the ability to efficiently integrate information across disparate regions via well-connected hubs. We find that the quality of patients' networks also correlates well with their degree of behavioural responsiveness, and some vegetative patients who show signs of hidden awareness have remarkably well-preserved networks similar to healthy adults. Overall, our research highlights distinctive network signatures of pathological unconsciousness, which could improve clinical assessment and help identify patients who are aware despite being uncommunicative.

## Introduction

There has been considerable recent interest in the view that consciousness is a phenomenon emerging from the dynamic equilibrium between differentiated and integrated information processing in the brain [1]–[4]. This view has inspired research into ways of quantifying the characteristics of information exchange in the brain at rest, and how this modulated in natural sleep, pharmacological sedation, and pathological coma and disorders of consciousness (DoC; including the vegetative and minimally conscious states, VS and MCS). In this latter case, such theoretical questions about the neural bases of consciousness take on a clinical and societal significance, as they could inform diagnosis, prognosis and treatment of DoC, which are often brought on by severe injury to the brain. Recent advances in the use of neuroimaging to better ascertain brain function in DoC have yielded some surprises, and indicated that a significant minority of patients are able to volitionally modulate brain activity in ways that would normally require high-level cognition and even covert awareness despite no behaviourally evident signs thereof [5]–[14].

Such findings have motivated parallel research into the study of brain connectivity in patients at rest, using MRI [15], [16], EEG [17]–[19] and TMS [20], [21] to derive surrogate measure of information integration and differentiation. Modern neuroimaging methods for assaying such connectivity, including Magnetic Resonance Imaging (MRI) and high-density electroencephalography (EEG), provide a surfeit of data that need to be reduced in dimensionality and coalesced into patterns to provide an overarching understanding of connectivity networks in the brain. Graph-theoretical analysis of such networks [22]–[24] has provided an elegant way to achieve this synthesis using resting state connectivity data [23]–[27] in sleep [28]–[31], sedation [32] and coma [33].

Here, we apply graph theory to extract patterns of information integration in brain networks derived from bedside measurement of high-density EEG in DoC patients, alongside normative networks observed in healthy controls. From 10 minutes of high-density EEG data, we calculate networks of sustained, coherent oscillatory activity within canonical frequency bands, which are prominent and commonly clinically evaluated in DoC. We will show that graph-theoretical metrics highlight contrasting *signatures* of connectivity in healthy and pathological brains across different frequency bands. These signatures, encompassing measures of topology as well as topography, will allow us to address a set of inter-related questions of fundamental neuroscientific importance: for example, what is distinctive about network dysfunction in pathological states of low awareness? To what extent are these network signatures consistent across patients? How do they correlate with the complexity of preserved behavioural responses? And perhaps most intriguingly, what network signatures can we observe in patients who seem behaviourally vegetative, but nevertheless demonstrate signs of covert awareness.

## Results

The findings described in this section are an exposition of the prominent changes to the spectral characteristics of resting state EEG in 32 DoC patients and 26 healthy controls. We begin with a description of changes in spectral *power* accompanying DoC, reiterating some well-established findings in the literature. These power-related changes are driven by fundamental alterations in the relative *amplitude* of ambient cortical oscillations commonly observed in the resting brain. We then move to novel analyses of changes in the structure of brain networks functionally unified by these oscillations. These changes are measured by spectral *connectivity*, which is derived from ongoing *phase* relationships between cortical oscillations.

### Spectral Power

**Figure 1:**
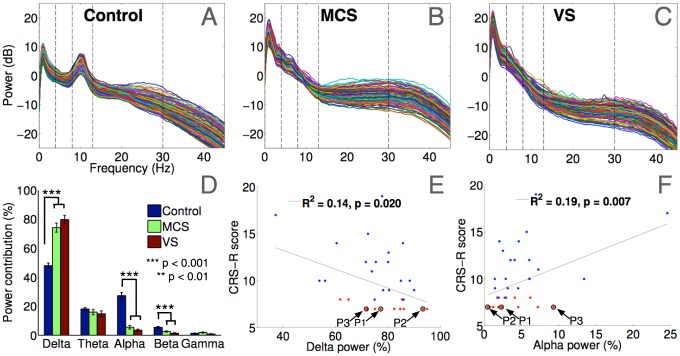
Band-wise power contributions in healthy controls and patients. Panels A–C depict mean channel-wise power spectra in controls, MCS and VS patients. Dashed lines indicated the boundaries of the canonical frequency bands: delta, theta, alpha, beta and gamma. Panel D plots the % contribution of power in each band to the total power in each channel, averaged across channels within each group. Panels E and F depict the opposite trends in power contributions from the delta and alpha bands of patients, as functions of their CRS-R scores. Patients P1, P2 and P3 are highlighted for comparison to plots in figure 6.

Figure 1A plots the log spectrum of each channel, averaged across 26 healthy controls. It generally conforms to the 1/f ‘pink noise’ decay that characterises human EEG, and is punctuated by prominent peaks in the canonical delta (0–4 Hz) and alpha (8–13 Hz) frequency bands. The topographic contributions of spectral power within these bands (see supplementary figure S1) shows that the power of the delta band peak is relatively more concentrated in frontal electrodes whereas the alpha peak is prominent in bilateral occipital electrodes. Across the spectra in the 3 groups in figures 1A–C, there was a prominent drop-off in alpha power in both MCS and VS patients, with a corresponding increase in delta power. This overall ‘slowing-down’ of resting state EEG, or slow-wave activity after severe brain injury has been documented [34]. To quantify this, we measured the channel-wise contribution to the average power across all channels within 0–40 Hz, from each frequency band. As expected, there were statistically significant differences in the relative power contributions from the delta, alpha and beta bands: as shown in figure 1D, patients together generated significantly more power in the delta band than healthy controls (Unequal variances t(55.7) = 9.97, p<0.001). In fact, 80% of overall spectral power in VS patients was concentrated within the delta band. The reverse was true in the alpha and beta bands: patients had significantly smaller power contributions from the alpha band (t(32.3) = 10.0, p<0.001). There was no significant difference in the power contribution from the theta band across the two groups. Visually, patient spectra in figures 1B and C suggested broadband increases in power in the higher i.e., beta and gamma frequency bands, due to elevated levels of electromyographic (EMG) noise introduced by involuntary muscle movements, especially in MCS patients. Hence, to avoid the consequent potential confounds in comparisons of activity in these bands between patients and healthy controls, we restricted ourselves to further analysis of data only within the alpha, theta and delta bands, where there were primary effects of interest and the influence of EMG noise was negligible.

While increased delta and theta power can sometimes be attributed to sleep-onset related EEG activity, we conventionally followed a protocol to ensure that our patients were arousable at the beginning of data acquisition (see Materials and Methods for details), which made it unlikely that they were consistently asleep during the following 10-minute period. We confirmed this by calculating the amount of temporal variability in power contributions within each frequency band in controls and patients. If patients were indeed falling asleep during the data acquisition, we would expect to see relatively higher variation in their delta band, alongside a progressive reduction in eye-movement related behaviour over the duration of the 10-minute recording. However, as can be seen in supplementary figure S2, there was no evidence of such higher variability: we found that healthy controls had higher temporal variations in delta (t(42.4) = 3.16, p = 0.003) and alpha (t(36.0) = 12.69, p<0.001) power. Further, we observed no consistently progressive decline in eye-movement related behaviour in patients, as recorded by derived electrooculographic (EOG) channels. As supplementary figure S3 depicts, though there was considerable variability in EOG activity between patients and over time, average activity in the first half of the recording session was statistically indistinguishable from the second half across patients (t(31) = 1.17, p = 0.25). This lack of a systematic decline suggested the absence of an unequivocal indication of sleep onset in the patient group.

While oscillatory power in the lower frequency bands derives from the predominance of so-called slow wave activity, alpha oscillations have been linked to arousal, attention and alertness [35]. Having observed significant group-level differences in power between patients and controls in these bands, we investigated the link between delta and alpha oscillations and clinically evidenced arousal in our patient group by quantifying the extent to which alpha power contributions could explain scores on the Coma Recovery Scale-Revised (CRS-R). The results of robust multilinear regression of delta and alpha power contributions as predictors of CRS-R scores are shown in figures 1E and F. As the regression lines plotted therein depict, there was a statistically significant link between a decrease in delta power, and a complementary increase in alpha power, with increase in CRS-R scores. This finding replicates the pattern recently reported by Lechinger et al. [36], who demonstrated a similar link between alpha power (and peak frequency) and CRS-R scores. In our data this trend was particularly visible in the MCS patient group (indicated in blue in figures 1E and F). These findings, taken together with previous evidence, are convergent with the notion that the presence of fast cortical oscillations is correlated with behavioural function in DoC.

### Spectral Connectivity

We assessed connectivity between EEG electrodes to investigate the structure of brain networks in the delta, theta and alpha bands. The extent of spectral coherence between every pair of electrodes was calculated using the *debiased weighted Phase Lag Index* (dwPLI) metric [37]. dwPLI is a sensitive measure of true connectivity between cortical regions that has been shown to be robust against the influence of volume conduction, uncorrelated noise, and inter-subject variations in sample size. An earlier incarnation of this measure, the *Phase Lag Index* [PLI, see 38], has been applied to low-density EEG acquired from DoC patients at rest, to show that those in VS elicit lower PLI values than MCS [18]. Here we calculated dwPLI for 91 channels in a high-density mesh (see Materials and Methods for details), to investigate the latent structure of connectivity networks that emerged within and across these groups.

**Figure 2:**
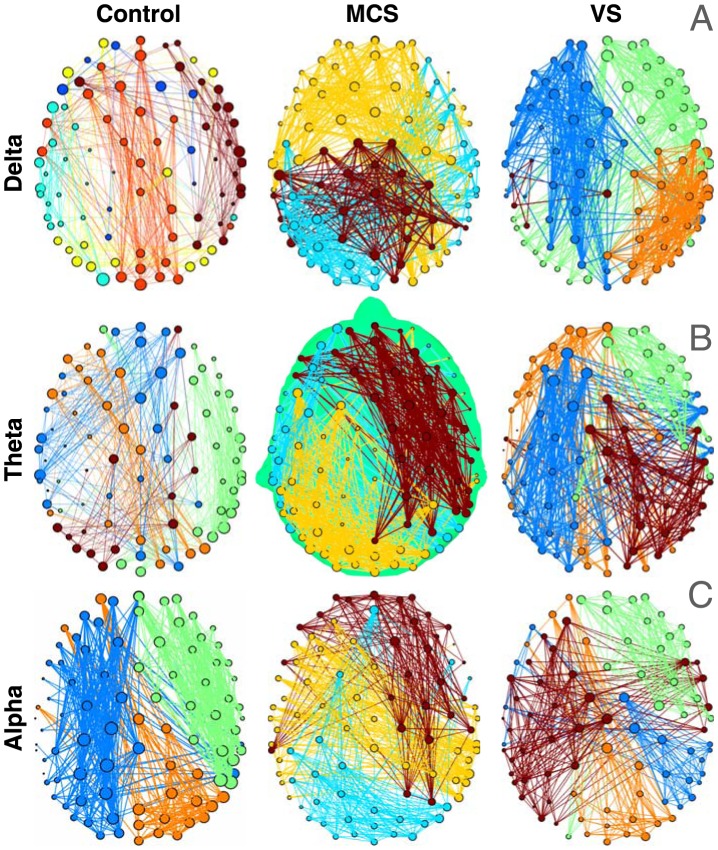
Band-wise connectivity networks in healthy controls and patients. Panels depict weighted connectivity networks averaged by group, for frequency bands of interest. In each network, the size of a node is proportional to its degree, and the thickness of an edge to its dwPLI weight. Modules identified by the Louvain algorithm are indicated by colour. For visual clarity, of the strongest 30% of edges, only the intra-modular edges are plotted. In the alpha band (panel C), healthy networks were characterised by a predominance of long-range frontoparietal modules. Patient alpha networks consisted of weaker, spatially localised modules. In contrast, patients had stronger structured connectivity within broadly synchronised modules in the delta and theta bands (panels A and B).

Figures 2A–C depicts topographic maps of dwPLI-derived connectivity for each group of subjects in each of the frequency bands of interest, visualising the opposing patterns of structure observed therein. To plot these maps, 91×91 dwPLI connectivity matrices were averaged within each group and plotted as topographs with electrodes as nodes and dwPLI values as edges, and graph-theoretical algorithms were employed to automatically identify modular structure therein. The topological structure of networks of alpha band connectivity in controls (see figure 2C, left) highlighted the presence of prominent modules (differentiated by colour) consisting of dense long-range synchrony that linked occipital, parietal and frontal electrodes. This structure was distinct from the bilateral, occipitally centered, distribution of alpha power of the scalp (see supplementary figure S1C, left), reinforcing the notion that dwPLI was measuring connectivity distinct from the effects of local volume conduction. It was also convergent with the notion that alpha networks observed over the healthy brain reflect broadly synchronous ambient cortical rhythms that are coeval with arousal and alertness. Such long-range connectivity structure in alpha connectivity was clearly lacking in patients, as is visually evident in figure 2C (middle and right) where a predominance of spatially localised, short-range synchrony was observed. Indeed, connectivity was generally weaker in patients, with mean dwPLI over all channel pairs significantly higher in controls (t(51.7) = 5.22, p<0.001).

**Figure 3:**
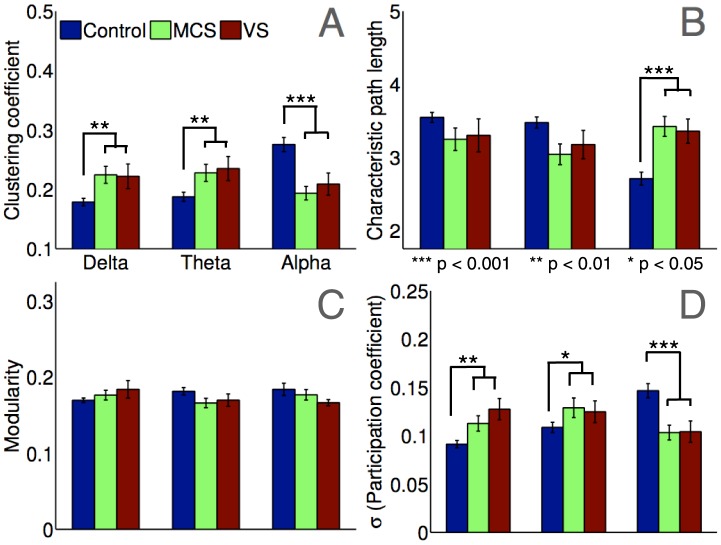
Graph-theoretic topology metrics of brain networks. Panels plot group-wise graph-theoretic metrics averaged over all connection densities considered. Clustering of alpha band networks in controls was significantly higher in controls than patients (Panel A), while characteristic path length was lower (Panel B). There were no significant differences in modularity between patients and controls in any frequency band (Panel C). SD of participation coefficients in control networks in the alpha band was significantly greater than patients, indicating the presence of diversely connected inter-modular hubs (Panel D). Differences in clustering and participation coefficient were markedly reversed in the delta and theta bands. Error bars indicate SE of the mean. All p-values were corrected for multiple comparisons.

Interestingly however, a contrasting pattern was evident in the delta and theta network topologies of the 3 groups, plotted in figures 3A and 3B: VS and MCS patients appeared to have relatively robust connectivity in the delta and theta bands. In particular, we observed the presence of prominent meso-scale modules in both VS and MCS patients. This suggested that the previously noted presence of higher power in the lower frequency bands in patients was concomitant with structured patterns of connectivity. Mean dwPLI of patients was also higher in the delta (t(40.3) = 2.82, p = 0.007) and theta (t(43.0) = 2.93, p = 0.005) bands. To quantify these structural differences in connectivity, we calculated and compared graph-theoretical summary measures of the topographs.

### Graph-Theoretic Network Metrics

The subject-wise dwPLI connectivity matrices in each band were thresholded at varying levels of connection density, to retain between 50–10% (step size of 2.5%) of the strongest dwPLI values in each matrix. At each value of this connection density threshold, we calculated 4 commonly measured metrics derived from graph theory, which captured topological properties of the networks observed in the thresholded connectivity matrices. Figure 3 plots the *clustering coefficient*, *characteristic path length*, *modularity*, and *participation coefficient* in each band and group, averaged across all connection densities considered. Supplementary plots the trends in these metrics as a function of decreasing connection density. These trends were generally consistent across the range of densities considered.

#### Clustering coefficient

The clustering coefficient of a network captures its *micro-scale* (local) efficiency [26], [39]. In line with the visual interpretation of figure 2C, we found significantly higher levels of clustering in alpha band networks of healthy controls compared to patients (figure 3A): t(52.0) = 4.86, p<0.001 (using an unpaired t-test with unequal variances; this p-value, and others reported later, are corrected for multiple comparisons across frequency bands using the Bonferroni-Holm correction [40]). However, the reverse was true in the delta and theta bands, with higher clustering, and hence local network efficiency, in patients (delta: t(46.6) = 3.36, p = 0.003; theta: t(51.6) = 3.09, p = 0.003).

#### Characteristic path length

In contrast to the clustering coefficient, the *macro-scale* characteristic path length measures the average topological distance between pairs of nodes in a graph [39], providing an indication of global efficiency. As shown in figure 3B, path lengths were much shorter in control alpha networks (t(55.8) = 5.03, p<0.001). Complimentarily, direct measurements of global efficiency (based on the average of inverse shortest path lengths [41]) in alpha networks also generated much higher values in controls than patients (t(54.8) = 4.86, p<0.001). However, the reverse was not true in the delta or theta bands of patients. In other words, despite the robust connectivity observed in patient networks within these lower frequency bands, they did not have the topological structure required to enable efficient macro-scale interactions across the cortex. We will later link this finding to topographical estimations of differences in scale using the modular span metric.

#### Modularity and participation coefficient

Modularity is a *meso-scale* network metric that encapsulates the degree to which the nodes of a network can be parcellated into densely connected, topologically distinct modules with relatively few inter-modular connections [42]. Given a modular decomposition, the participation coefficient is a inter-modular measure of network centrality, and flags up hub nodes that link many modules together in an efficient network [43]. As figure 3C shows, there were no significant differences in modularity between patients and controls. However, the standard deviations (SD) of participation coefficients of alpha network nodes were significantly higher (Figure 3D) (t(52.3) = 4.44, p<0.001), suggesting that the modules identified in healthy alpha networks were less segregated and more integrated, via diversely connected nodes that served as inter-modular hubs. Further, as is evident in figure 3D, this pattern was reversed in the lower frequency bands: the SD of participation coefficients in delta and theta band networks was significantly higher in patients (delta: t(49.0) = 3.63, p = 0.001; theta: t(53.2) = 2.03, p = 0.04).

#### Mutual information

**Figure 4:**
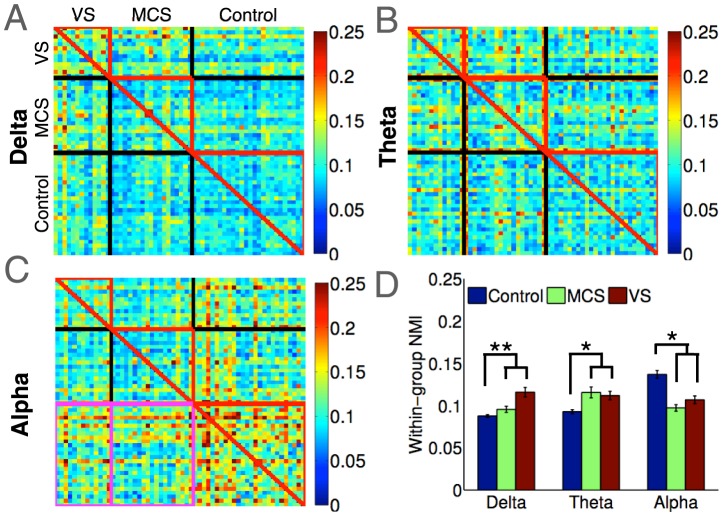
Normalised mutual information between brain networks. Panels A–C plot colour maps of individual NMI between networks of patients and controls. Red triangles encompass NMI values indexing degree of similarity of structure within each group. Within-group NMI between healthy alpha networks was significantly higher than that in patients (Panel D). This pattern was reversed in the delta and theta bands, where network structure was more similar between patients than controls. Magenta boxes in Figure 4C encompass between-group NMI in patients and healthy controls, correlated against their CRS-R scores in figure 6C.

Previous research into resting state networks involving patients in acute coma and chronic disorders of consciousness has reported on the disruption of structural and functional connectivity following brain injury [15], [16]. Recently, Achard et al. [33] employed graph-theoretic analysis of resting state fMRI scans and found that there was significant restructuring of network hubs in comatose patients. We investigated whether similar patterns could be observed in EEG networks in our group of DoC patients, using a normalised mutual information (NMI) metric [44]. Given a pair of networks, a high NMI value indicates that their modular decompositions are very similar, in that a node that belongs to a particular module in one network is likely to belong to the same module in the other. Achard et al. [33] used NMI to show that the modular structure of networks were more variable from one patient to the next, and also dissimilar to the relatively similar structures observed in healthy controls. We interrogated our data to examine this, and calculated the band-wise NMI between the module affiliations of every healthy control and patient. Figure 4C plots a colour map of these NMI values, where within-group values are highlighted by red triangles. As is evident by comparing them, there were much higher levels of similarity between healthy controls in modular structure of alpha band networks. As with fMRI networks in coma reported by Achard et al. [33], the modular structure of alpha band networks in DoC patients were neither consistently similar to healthy controls, nor to each other. We confirmed this statistically by comparing *within-group* alpha network NMI in healthy controls and patients, encompassing the values within the red triangles in figure 4C. As figure 4D shows, we found a statistically significant reduction in within-group alpha NMI in patients (t(50.8) = 2.32, p = 0.024). However, in contrast to previous findings indicating that brain networks in patients are variably disrupted [33], we found *higher* values of within-group NMI in their delta and theta band networks (red triangles in figures 4A and B). As shown in figure 4D, delta and theta band networks of patients had statistically *larger* within-group NMI than healthy controls (delta: t(44.3) = 3.28, p = 0.006; theta: t(45.1) = 2.96, p = 0.01). That is, modular structures of delta and theta band networks in patients were similar to each other, unlike in healthy controls.

#### Modular span

**Figure 5:**
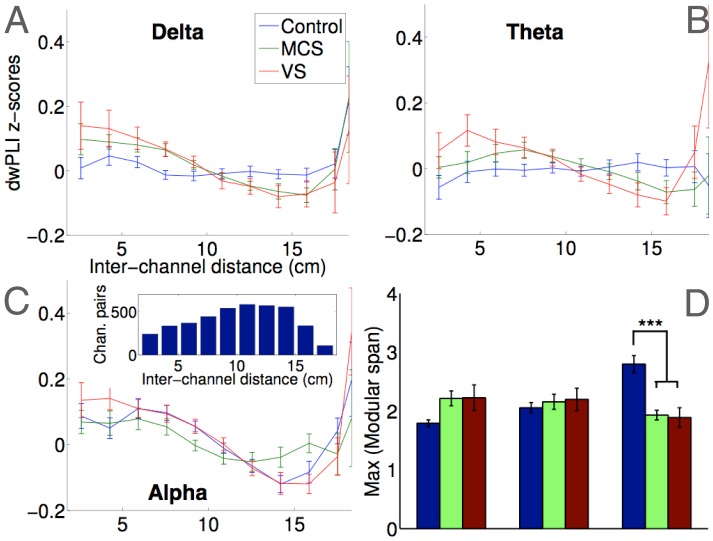
Topographical embedding of brain networks using modular span. Panels A–C plot group-wise averaged dwPLI values as a function of the Euclidean distance between pairs of EEG channels. Inset in Panel C plots a histogram of these inter-channel distances. Controls had stronger long-range connectivity in the alpha band, whereas patients had stronger local connectivity. Topographical distances spanned by alpha network modules, as measured by modular span, were significantly greater in controls (panel D). No differences were observed in modular span in other frequency bands.

While clustering, path length, modularity and participation coefficient quantify key *topological* characteristics of networks, they are by definition unaware of the *topographical* structure of the EEG networks considered here. To address this, we employed a novel network metric, *modular span*, which measured the average weighted topographical distance (over the scalp) spanned by a module identified in a network (see Materials and Methods section for details). Figures 5A–C plot band-wise dwPLI values as functions of the topographical distance between nodes (electrodes). They highlight the presence of stronger connectivity at greater distances in controls, whereas patients generally had more connectivity at short-to-medium topographical distances, as also reported by King et al. [19]. This pattern was quantified by the modular span metric: as figure 5D depicts, the modular span of the largest module in healthy alpha networks was much higher than patients (t(40.2) = 5.32, p<0.001), reflecting the presence of comparatively strong long-range connections in healthy brains (figure 2C, left). These were absent in patient alpha networks (figure 2C, middle and right), and resulted in greater topographical differentiation (i.e., spatial segregation) of modules, and consequently lower values of modular span. Interestingly, patient networks in the delta and theta bands did *not* elicit conversely higher modular span than healthy networks. This was in contrast to the topological measures considered above, which identified *greater* local efficiency and inter-modular centrality in delta and theta band networks in patients.

Taken together, the above comparisons of graph-theoretic metrics of networks provide quantitative support to the visual differences between the patterns of connectivity in patients and controls (see figure 2). On the whole, alpha band networks were more locally and globally efficient, less modularised and more interconnected in healthy controls than patients. Healthy alpha networks were also more similar to each other, and spanned greater distances to link distant regions. These findings are convergent with what we know about alpha band connectivity, and previous evidence showing disrupted alpha oscillations in DoC patients. However, our data also suggested the presence of significantly *higher* local efficiency, *lower* modularisation, and *higher* centrality in patient networks in the delta and theta bands. Further, compared to healthy networks, patient networks in these bands were also more similar to each other. Crucially however, we found a distinction between the opposing patterns uncovered by these topological metrics of the graphs and our topographic characterisation with modular span. As we elucidate further in the Discussion section, this distinction provides key insights into the nature of dysfunction in brain networks of patients.

### Clinical Correlations

**Figure 6:**
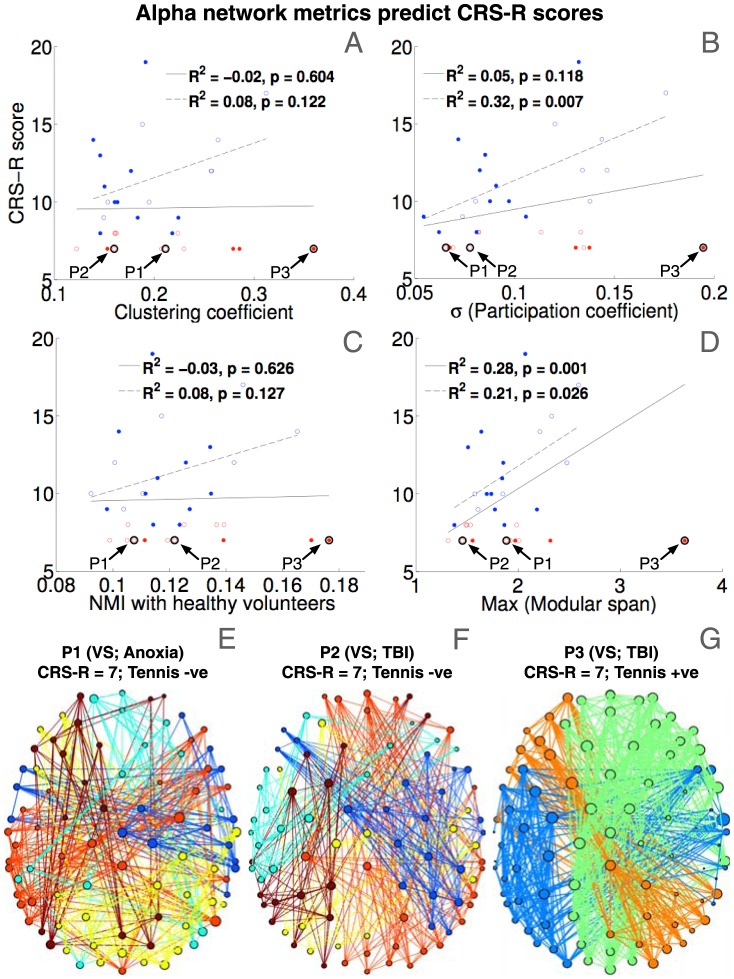
Graph-theoretic metrics as predictors of CRS-R scores. Panels A–D plot correlations between graph-theoretic metrics of alpha networks and behavioural CRS-R scores of individual patients. Red and blue circles indicate VS and MCS patients respectively. Filled circles indicate patients who followed command with fMRI tennis imagery. Robust linear regressions indicated by solid lines included all patients, whereas those indicated by dashed lines only included MCS patients. All metrics improved alongside progressive increase in CRS-R scores of MCS patients. Panels E, F and G plot alpha band networks of representative VS patients P1, P2 and P3, respectively. All 3 patients had the same CRS-R score, but only P3 showed evidence of command following. Compared to P1 and P2 (panels E and F), P3 also had remarkably well-preserved alpha network structure (panel G). Highlighted circles in panels A–D demonstrate that graph-theoretic metrics of P3's alpha network were exceptional outliers amongst the patient group, much more so than P3's delta/alpha power (see figures 1E and F).

Having established that there were consistent differences between EEG resting networks observed in patients and healthy controls, we set out to assess the link between graph-theoretic metrics derived from these networks and clinical evaluations of neurological and cognitive function in individual patients. As figures 3, 4 and 5 suggest, there were no prominent statistically significant differences between the metrics obtained in VS and MCS patients, potentially attributable to the nature of our convenience sample (see Discussion section). Focusing instead on the CRS-R scores of the patients, we noted that Lechinger et al. [36] recently showed a correlation between power ratios and peak frequency in the alpha band and CRS-R scores, a finding similarly observed in our data (see figure 1F). Following on from this, we first investigated whether mean dwPLI values of patient connectivity matrices were correlated with CRS-R scores. No significant correlation was found in any of the frequency bands of interest. Going beyond assessment of mean spectral power and connectivity, figures 6A–D plot the robust linear regressions of key graph-theoretic metrics of patient alpha networks, which significantly predicted CRS-R scores. Note that figure 6C plots average NMI between the alpha networks of a patient and each control (encompassing between-group values within the magenta boxes in figure 4C), indexing the degree to which a patient's network structure was like that of healthy brains.

There was a common pattern in the variation of these metrics that separated VS from MCS patients (plotted as red and blue circles, respectively). VS patients in our dataset, who by definition did not present much behavioural variation and were assigned CRS-R scores of either 7 or 8, nevertheless had considerable variation in the efficacy of their alpha band networks as measured by graph theoretical analysis. We found that some of this variation could be interpreted in light of independent evidence that some of the VS patients considered performed tennis imagery detected by fMRI (see Materials and Methods for details; also see Owen et al. [5] and Monti et al. [9]). The 4 VS patients who performed tennis imagery, indicated by filled circles in figures 6A–D, tended to be outliers in terms of the characteristics of their alpha band networks. As a representative example, the networks of three patients P1, P2 and P3, all with CRS-R scores of 7 but of whom only P3 performed tennis imagery, are shown in figures 6E, F and G. As is evident, alpha band connectivity in P3 was comparatively much better preserved, and was clearly distinct from the lack of structure in P2 or P3. Specifically, P3's alpha network, unlike P1's or P2's, was remarkably similar to healthy controls, and consisted of strong, long-range connections spanning occipital, parietal and frontal regions (compare figures 6E, F and G with figure 2C, left). In keeping with this visual interpretation, quantitative graph-theoretic metrics of P3's alpha band network, highlighted in figures 6A–D, were also exceptional compared to P1 and P2, and the rest of the VS patients. In particular, P3's alpha network had much higher local connectivity (figure 6A), more inter-modular hub nodes (figure 6B), and structurally similar modules to healthy controls (as measured by relative-to-healthy NMI, see figure 6C) that spanned greater topographical distances (as measured by modular span, see figure 6D). In particular, though these prominent differences between the brain networks of P1 and P3 could perhaps be attributed to aetiology, it could not explain away the differences between P2 and P3, as both had suffered traumatic brain injury. It is also interesting to note that though P3's alpha network properties were clearly very prominent outliers as compared to P1 and P2, delta and alpha power in P3 were much less exceptional (compare figures 6A–D to figures 1E and F). Hence characterising network signatures of spectral connectivity could considerably improve our understanding of residual brain function in behaviourally uncommunicative patients who nevertheless demonstrate covert awareness.

Among MCS patients, in whom there was more meaningful variability in CRS-R scores, we observed a clear trend toward increasing clustering, network centrality and modular span of alpha band networks as CRS-R scores improved (dashed regression lines in figures 6A–D). In other words, as the alpha networks of MCS patients approached levels of structured, long-range, inter-modular connectivity seen in healthy controls, their CRS-R scores got progressively higher. It is worth noting that the same regressions when including all patients (indicated by the solid lines) were much weaker due to the considerable variation of network metrics within the VS group in the absence of matching behavioural variation. We found that there were no significant correlations between graph metrics calculated from delta and theta band networks and CRS-R scores of patients, despite the significantly higher levels of topological connectivity observed in these networks (see figures 3 and 4). As we discuss next, this could potentially be explained by the limited topographical extent of connectivity in lower frequency bands. Overall, our results suggested that it was the structure of connectivity in the alpha band that generated the strongest link to behaviourally evidenced neurological and cognitive function in patients.

Finally, we examined whether there were systematic differences between graph-theoretic metrics of patients who evidenced covert awareness as measured by tennis imagery, vs. patients who did not. While none of the metrics of patients with positive imagery (filled circles in Figure 6) significantly differed from those of patients without such evidence, a key distinction was evident between the VS and MCS subgroups: as pointed out earlier, amongst the VS patients, those with evidence of positive imagery tended to have remarkably higher alpha graph-theoretic metrics. This difference could not be statistically verified due to the lack of power (only 4 out of 13 VS patients performed imagery). However, the pattern was reversed in MCS patients, where those with positive imagery tended to exhibit *lower* values of alpha metrics. In particular, inter-modular centrality (as measured by the SD of participation coefficients) was significantly lower in MCS patients who performed tennis imagery (t(10.8) = 2.93, p = 0.014), though modular spans (t(10.2) = 2.27, p = 0.046), clustering coefficients (t(9.5) = 2.19, p = 0.054) and characteristic path lengths (t(13.8) = 1.88, p = 0.081) were not. This somewhat paradoxical finding could potentially be explained by the observation (see figure 6) that MCS patients who did not perform tennis imagery also tended to score higher on the CRS-R scale, though their scores were not significantly higher. Hence it could be that these patients had progressed into a post-traumatic confusional state with potentially limited attention control, known to characterise emergence from MCS [14], .

## Discussion

The exploration of resting state EEG described above adds to convergent understanding of how structured connectivity in human brain networks is disrupted in DoC. Generally speaking, our graph-theoretical quantification showed that alpha networks in the healthy brain were balanced between *strong local interactions* (high clustering) and *robust interconnectivity* (more intermodular hubs). Such configurations in the alpha band were absent in patients, and provided a network-based account of the role of structured alpha connectivity in subserving arousal and awareness. This difference between patients and healthy controls is consistent with evidence from fMRI resting networks described by Vanhaudenhuyse et al. [15], who found reduced connectivity in the default mode network in patients, which has previously been linked to alpha power and synchrony [46]–[48]. Our findings with regard to alpha band networks are also consistent with another recent analysis of EEG networks in a large cohort of patients [19], which found a reduction in long-range information sharing in DoC. However, it should be noted that while we attempted to ensure that the group differences and correlations observed in the graph-theoretic measures cannot be explained away by systematic sleep onset in patients, considerations of ongoing and rapid fluctuations of arousal and vigilance in patients during the recording warrant careful interpretation of the limitations and generalisability of these measures, both within and across groups.

Despite observing robust differences between healthy controls and patients, the graph-theoretic metrics did not find any prominent statistically significant differences between our VS and MCS patient groups. Speculatively, the nature of our convenience sample could have contributed to this lack of a difference: VS patients included in our study had CRS-R scores between 7 and 8, close to the boundary between the VS and MCS states (see Table 1). Further, many of our MCS patients included had low to middle CRS-R scores in the 8–10 range. This CRS-R overlap between the two groups, in combination with insufficient statistical power due to the relatively limited number of patients in each group, could have blurred any differences between them. In comparison, King et al. [19] recently applied their novel weighted Symbolic Mutual Information (wSMI) connectivity measure to distinguish VS from MCS patients, albeit in a much larger group of 181 patients. Further, the CRS-R scores of their patients spanned a wider range, from 1–8 and 6–23 amongst VS and MCS patients, respectively, thereby sampling greater variability in connectivity networks. In this regard, a valuable future direction for this research would be to comparatively evaluate graph-theoretic network analytics based on potentially more sensitive connectivity measures like wSMI alongside other EEG markers [49].

**Table 1:** Demographic and assessment details of patients from whom EEG data was analysed.

Patient	Post-ictal Interval (months)	Gender	Age at Assessment (years)	Etiology	Diagnosis	CRS-R	Command Following (CRS-R)	Command Following (fMRI)
**P1**	8	M	36	Anoxia	VS	7	No	No
**P2**	4	M	26	TBI	VS	7	No	No
**P3**	4	M	23	TBI	VS	7	No	Yes
**P4**	29	M	31	TBI	VS	7	No	No
**P5**	21	M	24	TBI	VS	8	No	No
**P6**	7	M	57	Anoxia	VS	7	No	No
**P7**	6	F	24	TBI	VS	8	No	No
**P8**	46	M	48	TBI	VS	8	No	No
**P9**	3	M	22	Anoxia	VS	7	No	No
**P10**	8	M	36	TBI	VS	7	No	Yes
**P11**	3	M	53	TBI	VS	7	No	Yes
**P12**	13	F	35	TBI	VS	8	No	No
**P13**	12	M	20	TBI	VS	7	No	Yes
**P14**	22	M	17	Anoxia	MCS	10	Yes	Yes
**P15**	8	F	36	Anoxia	MCS	8	No	Yes
**P16**	12	F	60	TBI	MCS	12	Yes	Yes
**P17**	14	M	54	Anoxia	MCS	9	No	Yes
**P18**	8	F	31	Anoxia	MCS	10	No	No
**P19**	12	F	38	TBI	MCS	11	No	Yes
**P20**	21	M	66	TBI	MCS	10	No	No
**P21**	67	M	29	TBI	MCS	10	Yes	Yes
**P22**	4	M	19	TBI	MCS	12	No	No
**P23**	11	F	70	TBI	MCS	9	No	No
**P24**	6	F	30	Anoxia	MCS	9	No	Yes
**P25**	105	M	37	TBI	MCS	12	No	No
**P26**	15	M	38	TBI	MCS	8	No	Yes
**P27**	12	M	45	TBI	MCS	14	Yes	Yes
**P28**	79	F	26	TBI	MCS	15	Yes	No
**P29**	154	M	34	Anoxia	MCS	17	Yes	No
**P30**	3	M	24	TBI	MCS	13	Yes	Yes
**P31**	10	M	41	TBI	MCS	19	Yes	Yes
**P32**	7	M	38	TBI	MCS	14	Yes	No

However, despite the lack of group-level differences between our patients, we have shown that there were correlations between topological metrics of their alpha graphs and their clinical behaviours as measured by CRS-R scores: i.e., as patients' behaviourally evidenced function improved, so did the ‘quality’ and ‘normalcy’ of the topological characteristics of alpha networks. Our assessment of the *topographical* embedding to of these networks, using modular span, established that patient networks were also compromised in their ability to enable long-range connectivity in the alpha band. However, alongside the identification of damaged networks, our data highlighted the remarkable robustness of alpha connectivity in some behaviourally vegetative patients who also evinced high-level cognitive function by performing tennis imagery detected by fMRI. Hence, in such patients at least, we have found links between covert task-relevant attention and awareness, and the presence of brain networks that could support such advanced cognitive function despite the apparent lack of any consistent behavioural signs thereof. But as a group, more robust alpha networks were not predictive of positive tennis imagery in patients. While fundamental differences in the imaging modalities used to make these assessments could play a role in explaining such discrepancies, considerable arousal variation in patients during the intervening hours and days between these assessments also makes it difficult to unequivocally account for them. Further, differences in graph-theoretic metrics between patients with and without evidence of imagery were somewhat reversed in the VS and MCS groups. In particular, as pointed out earlier, MCS patients with relatively high CRS-R scores (in potential ‘confusional states’) and correspondingly robust alpha graph metrics tended *not* to show evidence of tennis imagery, highlighting the complexities inherent in correlating behavioural function with neuroimaging in DoC.

Complementary to findings in the alpha band, we have also highlighted the presence of higher levels of structured connectivity in the theta band in patients relative to healthy controls, as measured by the same topological measures. In the lower frequency bands, patient networks were more clustered, inter-connected and even similar to each other than were networks in healthy brains. Such increased power and connectivity in theta band has been reported in DoC [34], and attributed to layer V pyramidal neurons in partially deafferentated cortex, and the intrinsic tendency of such weakly interacting neuronal oscillators to synchronise [50]. In this context, our topographical evaluation of graph-theoretical network analysis identified a key distinction between brain networks in patients and controls: while alpha networks in controls were both topologically structured *and* topographically expansive, the topologically more robust delta/theta band networks in patients were *not* topographically expansive. In other words, despite being better inter-connected, patient networks in the delta and theta bands did not have significantly larger modular spans than healthy controls. This was in contrast to the reversed pattern in the alpha band, where the robust networks in healthy controls also had significantly larger modular spans. It is interesting to note here that administration of zolpidem to DoC patients temporarily can shift their theta band connectivity into the alpha and beta frequency bands [50], with potentially enhanced modular structure and spatial extent. Our analysis speak to this finding, providing insights into the characteristics of brain networks in DoC and reinforcing the link between observed network characteristics and underlying neurological dysfunction.

Finally, on a practical note, it is worth highlighting that short EEG recordings as analysed here are commonly measured in DoC patients in hospitals around the world, and clinically interpreted by eye by electrophysiologists. These could potentially become much more clinically informative if powerful analytical tools are used to unveil the capacity of cortical integration and differentiation, as captured by networks analyses such as those presented here. Combining easy-to-administer and inexpensive EEG with developments in network science could allow us to make inferences about information transfer across multiple scales of brain dynamics, and ultimately aid diagnosis and prognosis in this challenging group of patients.

### Conclusions

Our analysis of EEG connectivity in high-density networks at rest found that DoC patients had comparatively reduced graph-theoretic network efficiency in the alpha band as compared to healthy controls. Using a novel metric termed modular span that embedded topologically derived modules in topographical space, we established that the alpha network modules in patients were also spatially limited, with a prominent absence of the structured long-distance connectivity commonly observed in healthy networks. Importantly however, the observed differences between graph-theoretic metrics were partially reversed in the networks within the delta and theta bands. Here we noted the presence of robust connectivity patterns that were in fact commonly structured across patients, suggesting that there could be some degree of reorganisation, rather than just disorganisation, of brain networks in DoC. However, network modules in these lower bands did not have spatial spans that characterised healthy alpha modules. This finding addresses the question of why these lower band networks could not subserve balanced cortical integration and differentiation thought to be concomitant with normal consciousness. Going further, we found that alpha network metrics in patients clearly correlated with their behavioural scores on the CRS-R. Interestingly, we observed that some behaviourally vegetative patients who demonstrated evidence of command following with fMRI tennis imagery tended to deviate from this trend: their alpha networks were remarkably well preserved and were similar to those observed in healthy controls. On the whole, our findings describe distinctive signatures of brain networks in chronic disorders of consciousness. Further, in the significant minority of vegetative patients who show signs of covert awareness, they point to putative network mechanisms that could support high-level cognitive function despite behavioural impairment.

## Materials and Methods

### Ethics Statement

All healthy controls gave written informed consent. Ethical approval for testing healthy controls was provided by the Cambridge Psychology Research Ethics Committee (CPREC reference 2009.69) and the institutional ethics committee of the Faculty of Psychology of Universidad Diego Portales. Written informed consent was acquired from all patients' families and medical teams. Ethical approval for testing patients was provided by the National Research Ethics Service (National Health Service, UK; LREC reference 99/391). All clinical investigations were conducted in accordance with the *Declaration of Helsinki*.

### Participants

#### Healthy controls

A convenience sample of 26 neurologically healthy adults (14 male; 12 female) (mean age = 24.7; SD = 4.7) participated in the study.

#### Patients

A convenience sample of 34 VS or MCS patients, assessed at Addenbrooke's Hospital in Cambridge (UK) between January 2011 and July 2013 were included in the study. EEG data acquired from 2 patients were rejected due to excessive noise artefact. Demographic details of remaining 32 patients from whom data was analysed are listed in Table 1.

Patients were typically admitted for 4–5 days as part of a comprehensive testing protocol that included the EEG task described below, in addition to the fMRI tennis imagery task described by Owen et al. [5]. Patients were repeatedly assessed with the Coma Recovery Scale–Revised [CRS–R, 51] during their admission. As listed in Table 1, the highest CRS-R score observed across all assessments of each patient was used to assign a diagnosis of VS or MCS. Breakdowns of these scores according to the CRS-R subscales are listed in Table 2. Of the 32 patients, 13 were diagnosed to be VS, with highest CRS-R scores between 7 and 8. The 19 other patients diagnosed as MCS had a wider range of scores between 8 and 19.

**Table 2:** CRS-R subscores of patients.

Patient	Auditory	Visual	Motor	Oromotor	Communication	Arousal
**P1**	1	1	2	1	0	2
**P2**	1	1	2	1	0	2
**P3**	1	1	2	1	0	2
**P4**	1	1	2	1	0	2
**P5**	1	1	2	2	0	2
**P6**	1	1	2	1	0	2
**P7**	1	1	2	2	0	2
**P8**	1	1	2	2	0	2
**P9**	1	1	2	1	0	2
**P10**	1	1	2	1	0	2
**P11**	1	1	2	1	0	2
**P12**	2	1	2	1	0	2
**P13**	1	1	2	1	0	2
**P14**	3	1	2	2	0	2
**P15**	1	2	2	1	0	2
**P16**	3	3	2	2	0	2
**P17**	2	3	1	1	0	2
**P18**	1	3	2	2	0	2
**P19**	2	3	2	2	0	2
**P20**	1	3	3	1	0	2
**P21**	3	2	2	1	0	2
**P22**	2	3	3	2	0	2
**P23**	2	2	2	1	0	2
**P24**	1	3	2	1	0	2
**P25**	2	3	3	2	0	2
**P26**	1	2	2	1	0	2
**P27**	3	3	3	2	0	3
**P28**	3	4	4	1	0	3
**P29**	4	3	5	1	1	3
**P30**	3	5	2	1	0	2
**P31**	4	5	6	1	0	3
**P32**	3	4	4	1	0	2

### EEG Data Collection and Pre-processing

From each participant, we collected at least 10 minutes of 128-channel high-density EEG data in microvolts (uV), sampled at 250 Hz and referenced to the vertex, using the Net Amps 300 amplifier (Electrical Geodesics Inc., Oregon, USA). Resting state data from healthy controls were acquired in a state of relaxed *eyes-open* wakefulness, while fixating on a central cross to minimise eye movements. Eye-blink activity was visually evaluated to ensure that the controls had their eyes open throughout the 10-minute recording.

Data from patients was acquired with a consistent protocol that was conventionally employed to ensure that the patient had eyes open and was aroused at the beginning of data collection. In addition, data was collected with most patients in a sitting position, unless clinical circumstances necessitated otherwise, as previous research has shown that the supine position adversely affects arousal and behavioural responsiveness [52]. To objectively assess eyes-open/eyes-closed states, we measured eye-blink and eye-movement related activity in our data. To this end we derived left and right vertical bipolar electrooculographic (EOG) channels from our raw EEG data, as subtractions of channels 25 vs. 127, and 8 vs. 126, respectively. Similar to the approach employed by Cologan et al. [53] we filtered these derived channels with 1–3 Hz to focus on eye-movement related activity, and then calculated their standard deviations (SD) within a 1-second non-overlapping sliding window over time, normalised by the average SD over all such windows. Supplementary figure S3 plots the time course of this normalised SD for each patient, averaged over these two bipolar channels.

**Figure 7:**
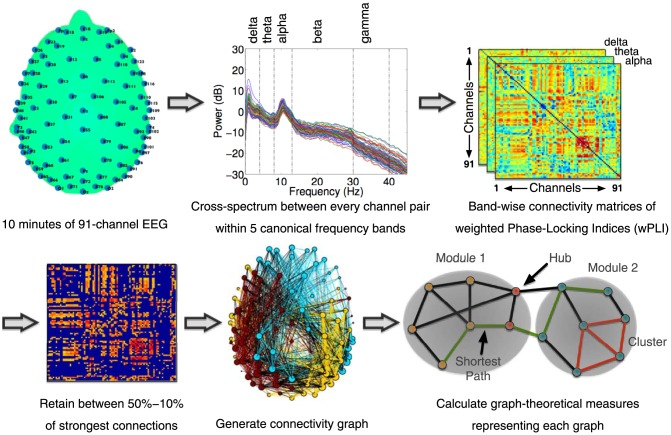
Data processing pipeline for graph-theoretical analysis. Cross-spectral density between pairs of channels was estimated using the dwPLI measure. Resulting symmetric connectivity matrices were thresholded before the estimation of graph-theoretic metrics. In the connectivity matrix shown (bottom left), the threshold has been set to plot top 30% of strongest connections. In the topograph (bottom middle), modules heuristically identified by the Louvain algorithm are indicated by colour, and inter-modular edges are plotted in black.

Data from 91 channels over the scalp surface (at locations shown in Figure 7, top left) were retained for further analysis. Channels on the neck, cheeks and forehead, which mostly contributed more movement-related noise than signal in patients, were excluded. Exactly 10 minutes of continuous data were retained, filtered between 0.5–45 Hz, and segmented into 60 10-second long epochs. Each epoch thus generated was baseline-corrected relative to the mean voltage over the entire epoch. Data containing excessive eye movement or muscular artefact were rejected by a quasi-automated procedure: abnormally noisy channels and epochs were identified by calculating their normalised variance and then manually rejected or retained by visual inspection. Independent Components Analysis (ICA) based on the Infomax ICA algorithm [54] was used to visually identify and reject noisy components. After pre-processing, a mean (SD) of 54 (7), 53 (7), 55 (2) epochs were retained for further analysis in VS, MCS patients and healthy controls, respectively. An ANOVA revealed no statistically significant difference between the numbers of epochs retained in the groups. Finally, previously rejected channels were interpolated using spherical spline interpolation, and data were re-referenced to the average of all channels. These processing steps were implemented using custom MATLAB scripts based on EEGLAB [55].

### Spectral Power, Connectivity and Graph-Theoretic Analysis

Figure 7 depicts the data processing pipeline employed to calculate spectral power and connectivity measures from the clean EEG datasets. Spectral power values within bins of 0.25 Hz were calculated using Fourier decomposition of data epochs using the *pwelch* method. At each channel, power values within five canonical frequency bands, *delta* (0–4 Hz), *theta* (4–8 Hz), *alpha* (8–13 Hz), *beta* (13–30 Hz) and *gamma* (30–40 Hz) were converted to relative percentage contributions to the total power over all five bands. Alongside, cross-spectrum between the time-frequency decompositions (at frequency bins of 0.49 Hz and time bins of 0.04 s) of every pair of channels was used to calculate a debiased, weighted *Phase Lag Index* (dwPLI) as introduced by Vinck et al. [37]. Generally speaking, phase synchronisation, widely seen as an EEG measure of information exchange between neuronal populations, is often calculated from the phase or the imaginary component of the complex cross-spectrum between the signals measured at a pair of channels. For example, the well-known Phase Locking Value (PLV; see Lachaux et al. [56]) is obtained by averaging the exponential magnitude of the imaginary component of the cross-spectrum. But many such phase coherence indices derived from EEG data are affected by the problem of volume conduction [57], [58], as a result of which a single dipolar source, rather than a pair of distinct interacting sources, can produce spurious coherence between spatially disparate EEG channels. The Phase Lag Index (PLI), first proposed by Stam et al. [38] attempts to minimise the impact of volume conduction and common sources inherent in EEG data, by averaging the signs of phase differences, thereby ignoring average phase differences of 0 or 180 degrees. This is based on the rationale that such phase differences are likely to be generated by volume conduction of single dipolar sources. But despite being insensitive to volume conduction, PLI has two important limitations: firstly, there is a strong discontinuity in the measure, which causes it to be maximally sensitive to noise; secondly, when calculated on small samples, PLI is biased towards strong coherences (i.e., it has a positive sample-size bias). The Weighted PLI measure (wPLI; see Vinck et al. [37]) addresses the former problem by weighting the signs of the imaginary components by their absolute magnitudes. The Debiased Weighted PLI (dwPLI) additionally addresses the latter problem by being minimally biased when the number of epochs is small. Further, as the calculation of wPLI also normalises the weighted sum of signs of the imaginary components by the average of their absolute magnitudes, it represents a dimensionless measure of connectivity that is not directly influenced by differences in spectral or cross-spectral power. For these reasons, we employed the dwPLI measure to estimate connectivity in our data.

For a particular channel pair and frequency band, the peak dwPLI across all time and frequency bins within that frequency band was recorded as the ambient amount of connectivity between those channels. Due to relatively higher levels of noise due to muscular artefact observed in patient spectra (see figure 1), this calculation of dwPLI-derived connectivity was restricted to the delta, alpha and theta bands, where the impact of such noise relatively negligible, and prominent differences between the power spectra were observed.

The 91×91 subject-wise, band-wise dwPLI connectivity matrices thus estimated were thresholded to retain between 50–10% of the largest dwPLI values. They were then represented as graphs with the electrodes as nodes and non-zero values as edges. The lowest threshold of 10% ensured that the average degree was not smaller than 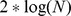, where *N* is the number of nodes in the network (i.e., *N* = 91). This lower boundary guaranteed that the resulting networks were estimable [39]. Similar ranges of graph *connection densities* have been shown to be the most sensitive to the estimation of ‘true’ topological structure therein [33], [59]: higher levels of connection density result in increasingly random graphs, while lower levels result in increasingly fragmented graphs.

At each step of the connection density between 50% and 10% in steps of 2.5%, the thresholded graphs were submitted to graph-theoretical algorithms implemented in the Brain Connectivity Toolbox [60]. These algorithms were employed to calculate metrics that captured key topological characteristics of the graphs at multiple scales. These included the micro-scale clustering coefficient and macro-scale characteristic path length [39] and global efficiency [41], alongside meso-scale measures like modularity and community structure [using the Louvain algorithm, see 61], and participation coefficient [43]. Modularity and community structure calculated by the heuristic Louvain algorithm, and all measures derived therefrom, were averaged over 50 repetitions. In addition, for each frequency band considered and at each connection density threshold, the normalised amount of mutual information [44] was calculated between the community structures in the graphs of each pair of subjects. Unlike some previous applications of graph theory to MRI data [33], [62], [63], we did not binarise the thresholded weighted graphs, to be able to better estimate path lengths and between-group differences therein [32], [64]. However, we verified that all the results described here, except those relating to characteristic path length, remained qualitatively unchanged when calculated with binarised matrices.

While the above graph-theoretic measures characterised the topological structure of networks, they did not capture how these networks were embedded in *topographical* space over scalp. To do this, we calculated a novel measure, termed *modular span*, which estimated the weighted topographical distance spanned by a module. More formally, given a thresholded graph with a previously identified community structure, the modular span *S* of a non-degenerate module *M* (i.e., a module with more than one member), was defined as:
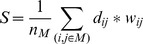
where *n_M_* is the number of nodes in the module, and (*i*, *j*) are a pair of member nodes therein. *d_ij_* is the normalised Euclidean distance between the pair of corresponding electrodes over the scalp, and *w_ij_* is the weight of the edge between nodes *i* and *j*. Note that, as *d_ij_* is the normalised distance (i.e., *d_ij_ = 1* for the most distant pair of electrodes), modular span is a dimensionless quantity. Modular span as defined above can be interpreted as the weighted sum of the topographic lengths of all the edges between the nodes comprising a module, scaled by the size of the module. By taking an algorithmically derived module of a graph and embedding it in the physical space over the scalp, modular span linked the topological construct with a topographical measure that provided key insights into the spatial differences between the brain networks of patients and controls.

We compared the graph metrics described above between groups of patients and controls in frequency bands of interest using unpaired t-tests, assuming unequal variances within the groups. The ability of the metrics derived from individual patient graphs to predict their CRS-R scores was tested using robust linear regression, by calculating R^2^ and p-values to estimate statistical significance.

## Supporting Information

Figure S1 **Group- and band-wise averaged topographic distributions of spectral power contributions.** Panels A, B and C depict topographic colour maps of group-wise power contributions to the delta, theta and alpha bands, respectively. Alpha power was primarily focused in occipital and parietal electrodes, whereas theta power was relatively frontocentral.
(EPS)

Figure S2 **Temporal variability in band-wise power contributions.** Bar graph depicts amount of variability in channel-wise power contribution percentages across epochs, band-wise averaged over all channels in each control and patient dataset. Patients generally had significantly lower or statistically indistinguishable amount of temporal variability in power contributions over the recording session.
(EPS)

Figure S3 **Temporal variability in EOG activity.** Time courses depict normalised standard deviations of derived electrooculographic (EOG) activity within 1–3 Hz, averaged over left and right EOG derivations, for each patient listed in Table 1.
(EPS)

Figure S4 **Graph-theoretic metrics as functions of connection density.** Panels A, B and C plot group-wise averaged graph-theoretic metrics in the delta, theta and alpha bands, respectively, as functions of decreasing connection density (increasing network sparseness). Error bars indicate SE of the mean. Differences between groups in these metrics were consistent across the range of connection densities considered.
(EPS)
